# Targeted senotherapy improves electrographic and behavioral outcomes in a mouse model of temporal lobe epilepsy

**DOI:** 10.1002/epi.70226

**Published:** 2026-04-13

**Authors:** David J. McFall, Abbas I. Hussain, Michelle Cho, Patrick A. Forcelli

**Affiliations:** ^1^ Interdisciplinary Program in Neuroscience Georgetown University Washington DC USA; ^2^ Department of Pharmacology and Physiology Georgetown University Washington DC USA

**Keywords:** epileptogenesis, hippocampus, senescence, SSK1

## Abstract

**Objective:**

Current pharmacotherapy for temporal lobe epilepsy (TLE) is limited to symptomatic treatment and leaves approximately one third of patients with inadequate seizure control. Discovering disease‐modifying targets is an unmet clinical need. We have previously identified senescent cells (SCs) as one such target. Many drugs that eliminate SCs (*senolytics*) interfere with apoptotic resistance proteins, potentially resulting in broad cytotoxicity and numerous side effects. Newer, more targeted therapies, like selective senescence killing compound 1 (SSK1), a gemcitabine prodrug that is selectively activated in SCs, offer the possibility to reduce off‐target effects, but SSK1 has yet to be investigated in any preclinical epilepsy model.

**Methods:**

We used pilocarpine to induce status epilepticus (SE) in 3‐ to 4‐month‐old mice. Immediately following SE, mice were randomly assigned to receive either SSK1 treatment or vehicle for the remainder of the study. We assessed behavioral performance on memory tasks, seizure burden by EEG, and histological markers of SCs.

**Results:**

SE robustly increased hippocampal and thalamic expression of SC marker p16 by over 100% compared to saline controls. SSK1 treatment reduced p16+ cells by ~45%, without any apparent neurotoxicity. In addition, SSK1 treatment normalized spatial memory impairments and reduced spontaneous seizure burden, completely protecting a majority (60%) of animals from seizures. SC burden in the hippocampus, but not the thalamus, correlated with seizure burden in vehicle‐treated animals.

**Significance:**

These findings lend further credence to the viability of targeting SCs to treat TLE. As with other genetic and pharmacologic SC ablation strategies, SSK1 produced a similar reduction in p16+ cells and normalization of spatial memory. SSK1, however, displays a stronger protective effect against seizures. In short, SSK1 is a compelling, translationally viable option for senolysis in TLE.

**Key points**

- Selective senescence killing compound 1 (SSK1) treatment removes senescent cells in the pilocarpine‐induced status epilepticus (SE) model.
- SSK1 treatment normalizes spatial and locational memory following SE.
- SSK1 treatment protected 60% of animals from developing epilepsy.
- Hippocampal senescent cell burden is tightly correlated with seizure burden.

## 1 INTRODUCTION

Despite considerable advances in pharmacotherapy, a third of patients with temporal lobe epilepsy (TLE) fail to achieve adequate seizure control.^1^ Closing this treatment gap is an important unmet clinical need. Current anti‐seizure medications suppress seizures but do not modify disease progression. Antiepileptogenic therapy, by contrast, would prevent the development of epilepsy after injury; no antiepileptogenic therapies exist for TLE.

Epileptogenesis is the result of a confluence of processes, including neuron loss, altered synaptic plasticity, aberrant neurogenesis, and chronic neuroinflammation.^2^ Cellular senescence, a conserved cellular program upregulated in response to stressors, has been shown to mediate many of these processes.^3^, ^4^, ^5^, ^6^, ^7^ Senescent cells (SCs) are identified by the expression of checkpoint inhibitor proteins p21^WAF/CIP1^ or p16^INK4A^, which lead to cell cycle arrest.^8^, ^9^ Cellular senescence is an essential process during both embryonic development and healthy wound healing, but following extreme insults, SCs accumulate faster than they can be cleared by resident immune cells and contribute to the inflammatory aspects of aging.^3^, ^10^, ^11^ SCs are increasingly recognized for their contribution to age‐associated disorders, including neurodegeneration, as removing these cells with genetic tools or SC ablating drugs (senolytics) improves outcomes.^6^, ^12^, ^13^, ^14^ Many factors secreted by SCs are implicated in epileptogenesis.^15^, ^16^

We recently reported that the removal of SCs improves behavioral outcomes and reduces seizure burden in the pilocarpine‐induced status epilepticus (SE) mouse model.^17^ We found similar benefits using a selective genetic approach (the INK‐ATTAC mouse model) and a well‐established senolytic cocktail of dasatinib (D) and quercetin (Q). The ATTAC strategy relies on expression of a transgene in SCs, and is therefore limited in its translation viability. D + Q has now been evaluated in a small number of clinical trials, and appears well tolerated.^18^, ^19^, ^20^, ^21^ Moreover, D is an U.S. Food and Drug Administration (FDA)–approved treatment for chronic myelogenous leukemia. However, D interacts with dozens of kinases (expressed both in SCs and in non‐SCs), modulates the immune system, and impacts chromatin structure in both SCs and young cells.^22^, ^23^, ^24^

To further validate and improve the translational potential of our previous findings, we tested the newly‐developed selective senescence killing compound 1 (SSK1), a galactose‐modified prodrug of gemcitabine.^25^ SCs robustly express ß‐galactosidase, which allows for conversion of the prodrug to gemcitabine selectively in SCs; this makes SSK1 a translationally compelling target for SC ablation.^25^, ^26^, ^27^, ^28^ We evaluated the effect of twice‐weekly SSK1 administration on the behavior, seizure burden, and histology of mice with chronic seizures following pilocarpine induced SE.

## 2 METHODS

### 2.1 Animals

Wild‐type male and female C57BL/6J mice (#000064) were obtained from Jackson Laboratory (Bar Harbor, ME, USA). Mice were socially housed in standard ventilated cage racks at a temperature of 22°C and a 12 h light–dark cycle (light: 0600–1800 h) with ad libitum access to food and water. All animal experiments and procedures were carried out in accordance with a protocol approved by Georgetown University's Animal Care and Use Committee (Protocol # 2019‐0037).

### 2.2 Status epilepticus

Three month to 4‐month‐old animals were pretreated with scopolamine methyl bromide (Sigma‐Aldrich, S8502) and terbutaline hemisulfate (Sigma‐Aldrich, T2528) (2 mg/kg, i.p.).^29^ Thirty minutes later, pilocarpine hydrochloride (280 mg/kg, i.p., Cayman Chemicals, C23131841) was administered. Animals were monitored behaviorally for acute seizures for 2 h immediately following pilocarpine administration using the following scale: (1) facial clonus, (2) unilateral forelimb clonus, (3) bilateral forelimb clonus, (4) rearing with forelimb clonus, (5) rearing with loss of balance, and (6) running and jumping. Two hours after pilocarpine administration, seizures were suppressed with diazepam (5 mg/kg, i.p., Dash Pharmaceuticals). Immediately following diazepam administration, mice were given dextrose solution (250 μL 5%, s.c.) to facilitate recovery. No SE (NSE) control mice received only scopolamine and terbutaline pretreatment.

### 2.3 Treatment

Animals were assigned randomly to treatment groups (SSK1 or VEH). Animals received their respective treatment immediately following diazepam administration. Mice received either SSK1 (.5 mg/kg, i.p., MedChemExpress HY‐138936) or a vehicle (90% phosphate buffered saline [PBS], 5% Tween‐80 [Sigma, P1754], 5% polyethylene glycol [Sigma, 81 172]) for two consecutive days every week for the duration of the study. This same concentration and dosing schedule was used previously used to eliminate SCs in young (3‐ to 6‐month‐old) mice following a pharmacologically induced lung injury.^25^

### 2.4 Behavior

Two months following SE, mice underwent a battery of behavioral tests. Following transport to the testing room, animals were acclimated to the testing room for 30 min before testing proceeded. Except where noted below, tests were performed in a 16″ × 16″ × 16″ plexiglass enclosure (TruScan Arena, Coulbourn Instruments, Whitehall, PA) under 20 lux of red light. Enclosures were cleaned with 70% ethanol between animals. ANY‐maze software was used for video tracking and supplementary analyses (distance traveled) (Stoelting Co., Wood Dale IL). Analyses were performed manually while blinded to treatment group.

#### 2.4.1 Open field test

Mice were placed in the plexiglass enclosure for 30 min to freely explore. The first 3 min were used to measure distance traveled and time in the center, defined as the time the animal's head and torso located within the central 25% of the enclosure.

#### 2.4.2 Novel Location Test and Novel Object Recognition Test

Both the Novel Location Test (NLT) and Novel Object Recognition Test (NORT) included habituation, familiarization, and probe phases. Three‐dimensional (3D)–printed shapes (cylinders, boxes, and pyramids) were used to ensure equal salience between objects. During familiarization, animals explored two identical objects (20 cm apart) for 5 min. After either a 5‐min delay in a separate cage or 24‐h delay in their standard cage, mice returned to the chamber where one object was either moved 20 cm toward the opposite wall (NLT) or replaced with an unfamiliar object (NORT). Animals explored for 5 min during probe phases. Preference index was calculated as time exploring the novel/moved object divided by total exploration time. Exploration was defined as when a subject's head was both oriented toward an object and within 1 cm of the object. Climbing was not considered interaction. Only animals with ≥10 s total object interaction during familiarization were analyzed.

#### 2.4.3 Object Context Mismatch Test

The Object Context Mismatch (OCM) test utilized two distinct chambers: the previously used square plexiglass enclosure and a covered circular plexiglass enclosure (10″ diameter, 8″ tall, Pinnacle Tech, 8228) with bedding. Animals were habituated to the enclosure for 2 days without objects (10 min in each enclosure). On the third day, animals were placed in the circular enclosure. After 5 min, they were transferred to the square enclosure for 5 min. Each chamber contained a unique pair of objects (e.g., two cubes in the square chamber; two cylinders in the round chamber). Following familiarization, mice were placed a standard housing cage for 5 min. To probe object–context mismatch memory, animals were returned to the circular chamber with one of the objects replaced with an object from the square chamber. Mice were given 3 min to explore the objects. Preference ratio was defined for the “context mismatched” object (e.g., the cube in the circular enclosure) using the same parameters and exclusion criteria as the NOL and NORT described.

#### 2.4.4 Elevated plus maze

Mice were tested in a standard elevated plus maze (EPM; San Diego Instruments) elevated 15″ above the ground. At the begin of testing, mice were placed in the central junction of the four arms. Tests lasted for 3 min, where the time an animal's head entered either of the open arms was recorded. If an animal fell off the apparatus during the test, it was retested the following day. If the animal fell during the retest, it was excluded from analysis.

#### 2.4.5 Barnes maze

Animals were tested using a mouse Barnes maze (BM) (92 cm diameter with 20, 5 cm diameter holes, evenly spaced along the perimeter of the maze; Maze Engineers). The maze was brightly lit (900 lux overhead lighting) with moderate levels of radio static (85 dB radio static) to motivate the animal to escape. Escape box location and spatial cues were kept constant throughout testing. Testing consisted of five consecutive days, with four trials per day (each trial lasting a maximum of 180 s) and a 15 min intertrial interval. At the end of a trial, animals were left in the escape box for 30 s before being returned to their cage.

### 2.5 EEG surgery

Three and a half months after SE, animals were anesthetized via 1%–3% inhaled isoflurane (FirstVet) and placed in a stereotaxic frame with blunt ear bars. Following standard surgical prep (shaving and iodine and alcohol swabs), .05 mL of .25% bupivacaine was administered to the incision site (s.c.) before making a roughly 1 cm midline incision in the scalp. Wireless EEG telemeters (EMKA easyTEL S) were inserted to the dorsum of the animal after making a subcutaneous pocket. Two craniotomy holes were placed using a pin vise with a 1 mm drill bit. Insulated stainless steel epidural electrodes were then placed bilaterally over the parietal cortex and fixed in place with dental cement (Stoelting, 10‐000‐786). The incision was closed with 3‐0 or 4‐0 absorbable sutures in a simple interrupted pattern. The surgical site was treated with triple antibiotic ointment. Animals were given a bolus of .5 mL saline and carprofen (5 mg/kg, both s.c.) and recovered in a clean cage with a heating pad. Following surgery, animals were housed individually for the remainder of the study.

### 2.6 EEG monitoring and analysis

Data were acquired via a DSI MX2 data exchange matrix, PhysioTel RPC‐1 receivers, and LabChart bridge software, and stored for offline analysis. Animals were recorded continuously for 11 days, beginning ~4 months after SE. Data were imported into an in‐house MATLAB processing pipeline (https://github.com/forcelli‐lab) for seizure detection as described previously.^17^ Briefly, we used a four‐pass strategy, using line length and frequency of peaks to identify putative seizure events. Features of these events (power, skewness, kurtosis, duration, etc.) were used to generate a T‐SNE plot for manual review. Retained events were a minimum of 5 s long and displayed evolution of shape and amplitude over time. Retained events were reviewed individually with the ability to adjust the boundaries of the seizure event. For each animal we calculated the (1) total number of seizures; (2) the average seizure duration, which was defined as the average of the lengths of individual seizures during the 11 day period; and (3) the cumulative seizure duration, which was the total duration of electrographic seizure activity, reflecting a combined metric of both seizure number and seizure duration. Seizures were scored by D.J.M. and reviewed by P.A.F.

### 2.7 Immunofluorescence

Mice were anesthetized via .2 mL/kg of Euthasol (sodium pentobarbital and sodium phenytoin solution) and perfused transcardially with 10 mL ice cold 1× Phosphate Buffered Saline (PBS) followed by 5 mL 4% paraformaldehyde (PFA) in PBS. Brains were removed and postfixed in 4% PFA overnight, and then cryoprotected in 30% sucrose in .1 M PBS. Brains were flash frozen in isopentane and sectioned at 25 μm thickness on a cryostat. Slides were washed 3× with 1× PBS + .1% Tween‐20 (5 min each), and then blocked with a solution of .3 M glycine, 2% bovine serum albumin, and 5% normal goat serum in 1 x PBS + .1% Tween‐20 for 90 min at room temperature. Mouse‐on‐Mouse blocking (Vector Labs) was performed for 1 h when using mouse primary antibodies. Primary antibodies were incubated overnight at 4°C. The following primary antibodies were used: mouse anti‐p16 (monoclonal, 1:500, Abcam, Ab54210), rabbit anti‐Iba1 (polyclonal, 1:500, Wako 019‐19741), and rabbit anti‐NeuN (monoclonal, 1:500, Invitrogen 702022). After washing, Alexa Fluor secondary antibodies (1:1000) were applied for 1 h at room temperature. When visualizing p16, anti‐IgG2b cross‐adsorbed secondary was used to minimize nonspecific binding (Invitrogen, A‐21145). Slides were washed, quenched for autofluorescence using Vector TrueVIEW Quenching Kit (Vector Labs), and mounted with Vectashield with 4',6‐diamidino‐2‐phenylindole (DAPI) before fluorescence microscopy.

### 2.8 Microscopy and image analysis

Widefield fluorescence images (20×) were acquired using a Mica Confocal Workstation (Leica). The entire hippocampus within a coronal section was outlined and imaged; Z projections were processed using Leica's computational clearing workflow and mosaics were exported for analysis in QuPath,^30^ where cell segmentation and object classification were used to analyze images. Ten to 15 representative training images were manually notated for the classifier before being saved as a scoring algorithm for batch analysis. At least three regions were evaluated per animal.

### 2.9 Statistics

All data are expressed as mean ± standard error of the mean (SEM). Wherever possible, data were collected and analyzed with the evaluator/s blinded to treatment group. GraphPad Prism (v.10.6.1) was used for statistical analysis. Histological and behavioral data were analyzed by Kruskal–Wallis tests with Dunn's post hoc test. BM data were analyzed by two‐way analysis of variance (ANOVA). Seizure data were analyzed by Mann–Whitney *U* tests and Fisher's exact test. Spearman's *r* was used to correlate daily seizure burden with SC burden.

## 3 RESULTS

### 3.1 SSK1 treatment reduces p16+ cells, but not neurons, following SE

**FIGURE 1:**
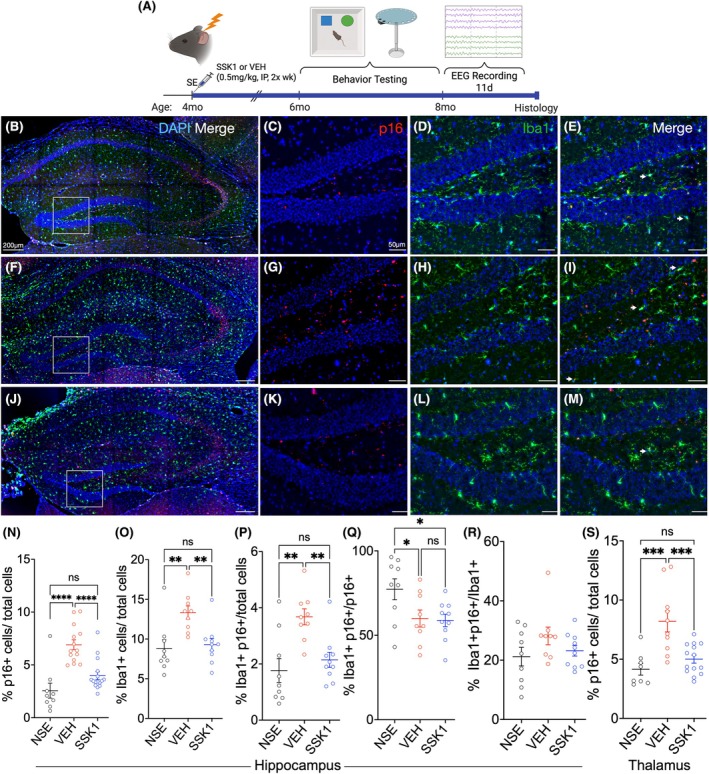
Selective Senescence Killing Compound 1 (SSK1) treatment reduces relative p16+ cell abundance in the hippocampus and thalamus following status epilepticus (SE) in mice. (A) Experimental overview. Three‐month to —4‐month‐old male and female C57BL/6J receive either vehicle (VEH) or SSK1 treatment two consecutive days a week, every week for the duration of the study. Two months after SE mice were tested for behavioral phenotypes, and 3.5 months after SE mice were implanted with wireless telemeters for 11 days of electroencephalographic (EEG) monitoring beginning at 4 months post SE. Following EEG monitoring, brains were prepared for histology. (B–M) Representative images of p16 (red) and Iba1 (green) expression in No SE (NSE) (B–E), vehicle‐ (F–I), and SSK1‐treated (J–M) mice 4 months after SE. (N) SE significantly increases hippocampal p16+ cells in VEH, but not SSK1, mice (*H* = 20.74, *p* < .0001; NSE vs VEH: *p* < .0001, NSE vs SSK1: *p* = .2544, VEH vs SSK1: *p* = .0051; Kruskal–Wallis with Dunn's post‐test). (O) SSK1 treatment normalizes SE‐induced increase in microglia (*H* = 11.56, *p* = .0031; NSE vs VEH: *p* = .0054, NSE vs SSK1: *p* > .9999, VEH vs SSK1: *p* = .0176; Kruskal–Wallis with Dunn's post‐test). (P) SSK1 treatment normalizes SE‐induced increase in p16+ microglia (*H* = 11.13, *p* = .0038; NSE vs VEH: *p* = .0040, NSE vs SSK1: *p* > .9999, VEH vs SSK1: *p* = .0455; Kruskal–Wallis with Dunn's post‐test). (Q) SSK1 treatment does not affect the relative proportion of SCs that are microglia following SE (*H* = 5.699, *p* = .0579; Kruskal–Wallis test). (R) The proportion of microglia that are senescent does not change following SE or SSK1 treatment (*H* = 2.145, *p* = .3422; Kruskal–Wallis test). (S) SE significantly increases thalamic p16+ cells in VEH, but not SSK1, mice (*H* = 13.24, *p* = .0013; NSE vs VEH: *p* = .0016, NSE vs SSK1: *p* = .7651, VEH vs SSK1: *p* = .0184; Kruskal–Wallis with Dunn's post‐test). Scale bars: (B, F, J) = 200 μm, (C–E, G–I, K–M) = 50 μm. N: NSE (*n* = 9), VEH (*n* = 14), SSK1 (*n* = 16). O–R: NSE (*n* = 9), VEH (*n* = 9), SSK1 (*n* = 10). S: NSE (*n* = 8), VEH (*n* = 10), SSK1 (*n* = 14). Mean ± SEM.

**FIGURE 2:**
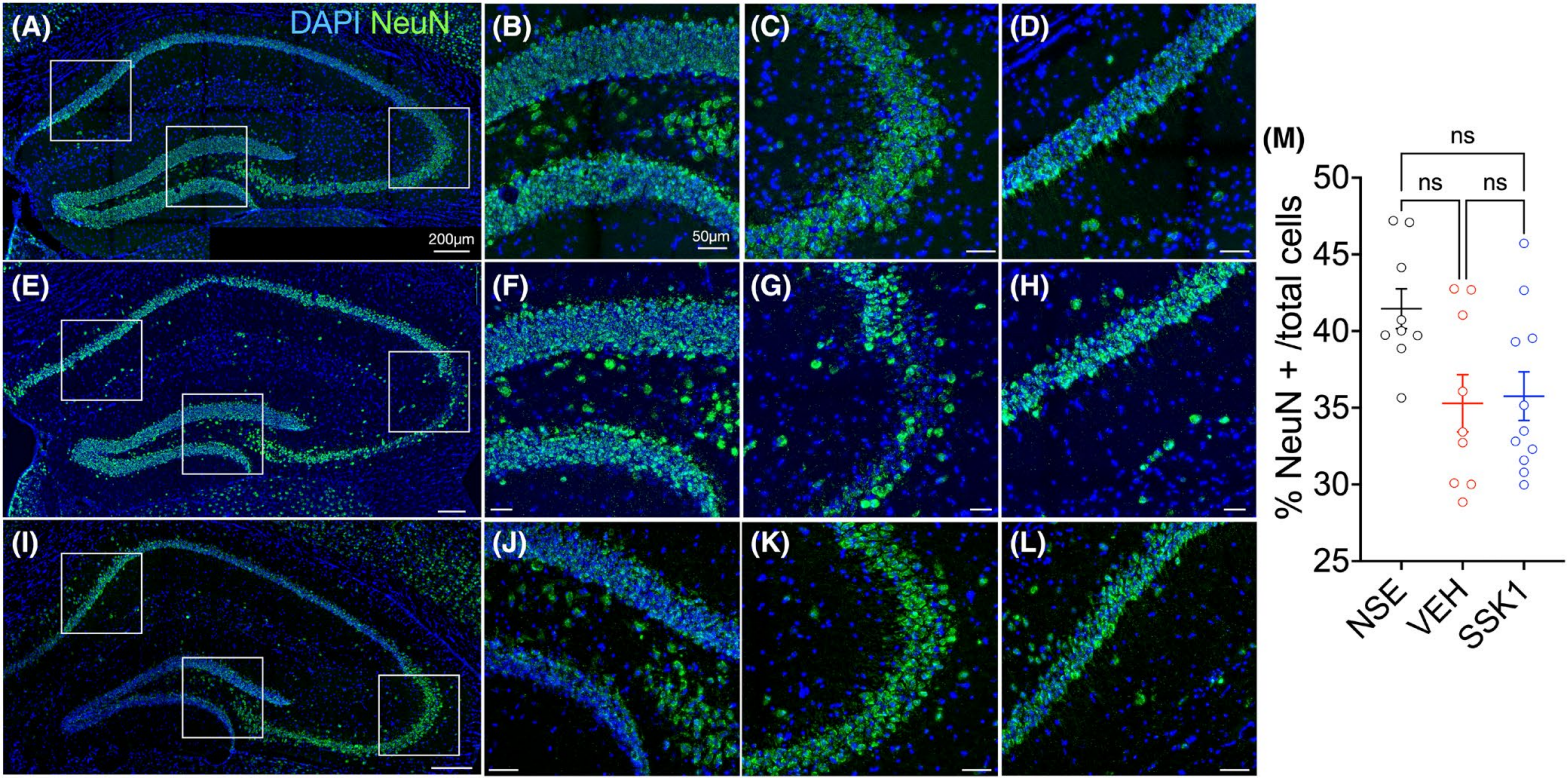
Selective senscence killing compound 1 (SSK1) treatment does not affect NeuN expression in the hippocampus. (A–L) Representative images of hippocampal NeuN (green) expression in No SE (NSE) (A–D), vehicle‐ (VEH; E–H), and SSK1‐treated (I–L) mice 4 months after SE. (M) SE significantly reduces hippocampal NeuN expression in VEH and SSK1 mice, with no significant difference between the two treatment groups (*H* = 6.480, *p* = .0392 NSE vs VEH: *p* = .0925, NSE vs SSK1: *p* = .0678, VEH vs SSK1: *p* > .9999; Kruskal–Wallis with Dunn's post‐test). Scale bars: (A, E, I) = 200 μm, (C,D, F–H, J–L) = 50 μm. M: NSE (*n* = 9), VEH (*n* = 9), SSK1 (*n* = 11). Mean ± SEM. NeuN, Neuronal nuclear antigen.

To determine the efficacy of SSK1 treatment to remove SCs, limit behavior deficits, and prevent epileptogenesis, we used the pilocarpine‐induced SE mouse model and compared three groups: NSE (no status epilepticus), SE + VEH, and SE + SSK1. As expected, the SE + VEH group displayed a significant increase in both the number of microglia and number of p16+ SCs, as compared to the NSE group (Figure 1N,O). This is consistent with our prior report that p16 expression is the primary SC marker observed after SE, and that it is particularly enriched in microglia.^17^, ^31^ SE + SSK1‐treated mice had on average ~45% fewer hippocampal SCs compared to SE + VEH mice; the SE + SSK1 group did not differ significantly from the NSE group (Figure 1N). Similarly, the SE + SSK1 group had fewer microglia in the hippocampus compared to SE + VEH mice, and did not differ from the NSE group (Figure 1O). Across groups, the majority of the SCs in the hippocampus were microglia (Figure 1Q). SSK1 treatment reduced the number of these double‐positive cells again by ~45% (Figure 1P), without affecting the relative proportion of p16+ microglia (Figure 1R). We saw a similar pattern of p16 expression in the rostral thalamus, with a significant increase in SE + VEH mice over NSE mice, which was normalized in the SE + SSK1 group (Figure 1S). We also stained the hippocampus for NeuN (neuronal nuclei antigen; Fox‐3). The SE + VEH and SE + SSK1 groups did not differ from each other, but both displayed a non‐significant trend (~15% reduction) in NeuN+ cells compared to NSE mice (Figure 2M).

### 3.2 SSK1 treatment alleviates spatial memory deficits following SE

**FIGURE 3:**
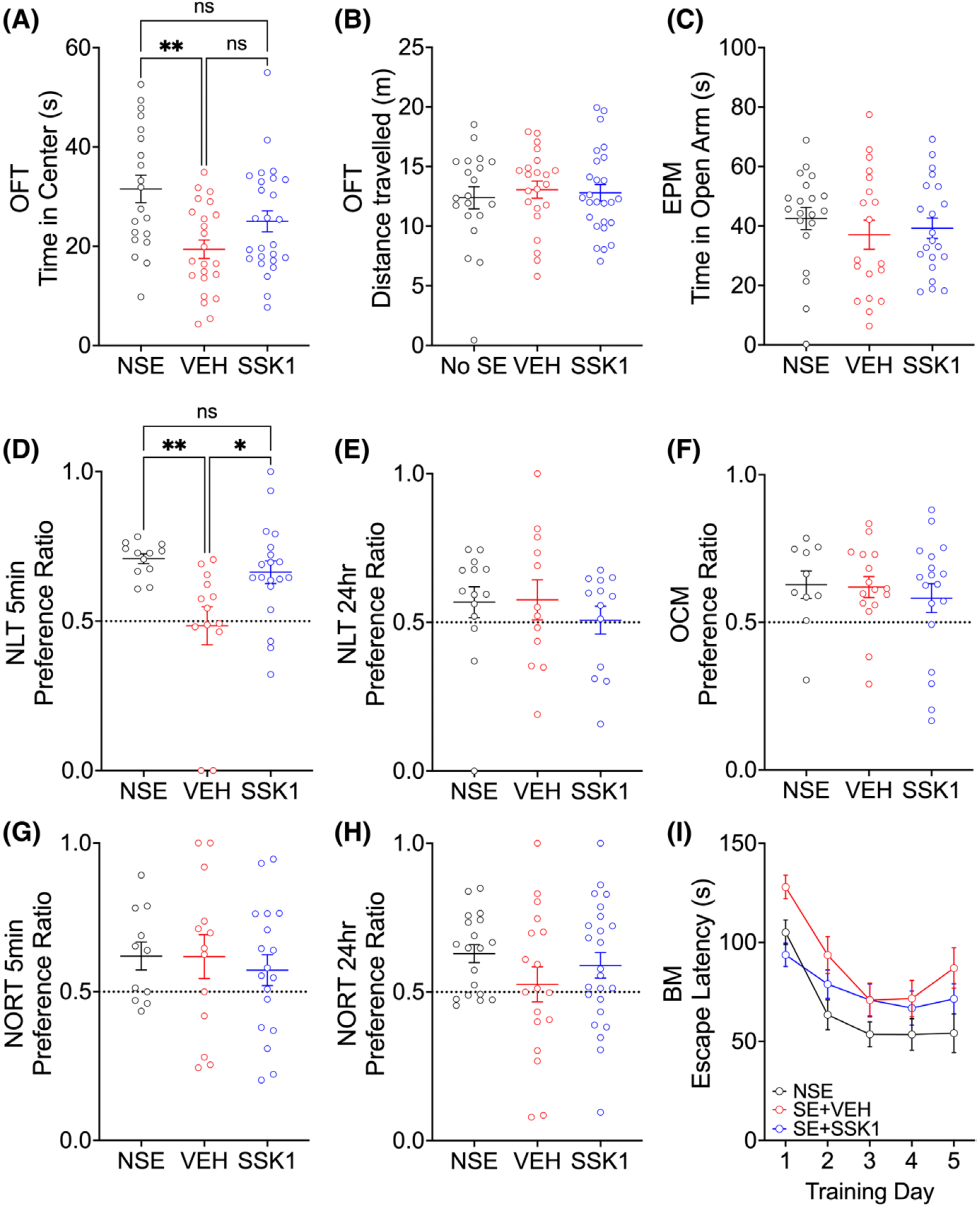
Selective senescence killing compound 1 (SSK1) improves spatial memory, but not object memory or anxiety‐like behavior, following status epilepticus (SE). (A) SE significantly reduces time spent in the center of the of the Open Field in vehicle‐treated (VEH) mice. SSK1 does not significantly differ from either no status epilepticus controls (NSE) or VEH groups (H = 11.04, *p* = .0040; NSE vs VEH: *p* = .0027, NSE vs SSK1: *p* = .2637, VEH vs SSK1: *p* = .2287; Kruskal–Wallis with Dunn's post‐test). (B) Neither SE nor SSK1 treatment affected distance traveled in the first 3 min of the OFT (*H* = .4037, *p* = .8172; Kruskal–Wallis test). (C) Neither SE nor SSK1 treatment significantly affected the amount of time spent in the open arms of the elevated plus maze (*H* = 1.117, *p* = .5721; Kruskal–Wallis test). (D) SSK1 treatment normalized novel location preference deficits seen in VEH mice in the 5 min delay NLT (*H* = 12.26, *p* = .022; NSE vs VEH: *P* = .0019, NSE vs SSK1: *P* = .5883, VEH vs SSK1: *P* = .0400; Kruskal–Wallis with Dunn's post‐test). (E) No differences were observed in the 24 h delay NLT (*H* = 1.505, *p* = .4711; Kruskal–Wallis test). (F) No differences were observed in the object context mismatch test (*H* = .2097, *p* = .9005; Kruskal–Wallis test). (G) No differences were observed in the 5 min delay NORT (*H* = .3420, *p* = .8428; Kruskal–Wallis test). (H) No differences were observed in the 24 h delay NORT (*H* = 1.821, *p* = .4022; Kruskal–Wallis test). (I) SSK1 treatment in SE mice improved navigational learning in the Barnes maze compared to VEH mice (Main Effect of Treatment: *F*_2,63_ = 4.436, *p* = .0158). We also observed the expected Main Effect of Trial Day: (*F*_3.788,238.6_ = 23.15, *p* = <.0001), but no Interaction Effect: (*F*_7.576,238.6_ = 1.505; *p* = .1603). A: NSE (*n* = 20), VEH (*n* = 23), SSK1 (*n* = 26). B: NSE (*n* = 20), VEH (*n* = 22), SSK1 (*n* = 26). C: NSE (*n* = 20), VEH (*n* = 19), SSK1 (*n* = 21). D: NSE (*n* = 12), VEH (*n* = 13), SSK1 (*n* = 19). E: NSE (*n* = 14), VEH (*n* = 12), SSK1 (*n* = 13). F: NSE (*n* = 10), VEH (*n* = 16), SSK1 (*n* = 18). G: NSE (*n* = 11), VEH (*n* = 13), SSK1 (*n* = 18). H: NSE (*n* = 19), VEH (*n* = 18), SSK1 (*n* = 24). I: NSE (*n* = 18), VEH (*n* = 22), SSK1 (*n* = 26). Mean ± SEM.

We reported previously that senolytic treatment rescues spatial memory deficits, but not anxiety‐like behaviors, following SE.^32^, ^33^ To assess anxiety‐like behaviors, we used the open field test (OFT) and the EPM. In the OFT, SE + VEH mice spent significantly less time in the center zone in the first 3 min compared to NSE mice (Figure 3A). SSK1 treatment did not result in statistically significant changes in time in the center zone compared to either the NSE or the SE + VEH groups (Figure 3A). Distance traveled in the OFT did not differ between groups (Figure 3B). In the EPM, we did not observe any significant differences when measuring the time spent in the open arms (Figure 3C).

To test different aspects of memory, we used the NLT, NORT, OCM, and BM. In the NLT, SE + VEH mice had a significantly lower novel preference ratio in the 5‐min recall test compared to NSE mice, consistent with the expected impairment in object location memory seen after SE (Figure 3D). SE + SSK1‐treated mice interacted with the object in the novel location for significantly longer compared to SE + VEH mice and did not differ from NSE mice (Figure 3D). The NSE, SE + VEH, and SE + SSK1 groups did not differ in the 24 h recall for this test, the OCM test, or the NORT (Figure 3E–H). In the BM, SE + VEH animals displayed delayed acquisition, which was alleviated by SSK1 treatment (Figure 3I).

### 3.3 SSK1 treatment reduces seizure burden in SE mice

**FIGURE 4:**
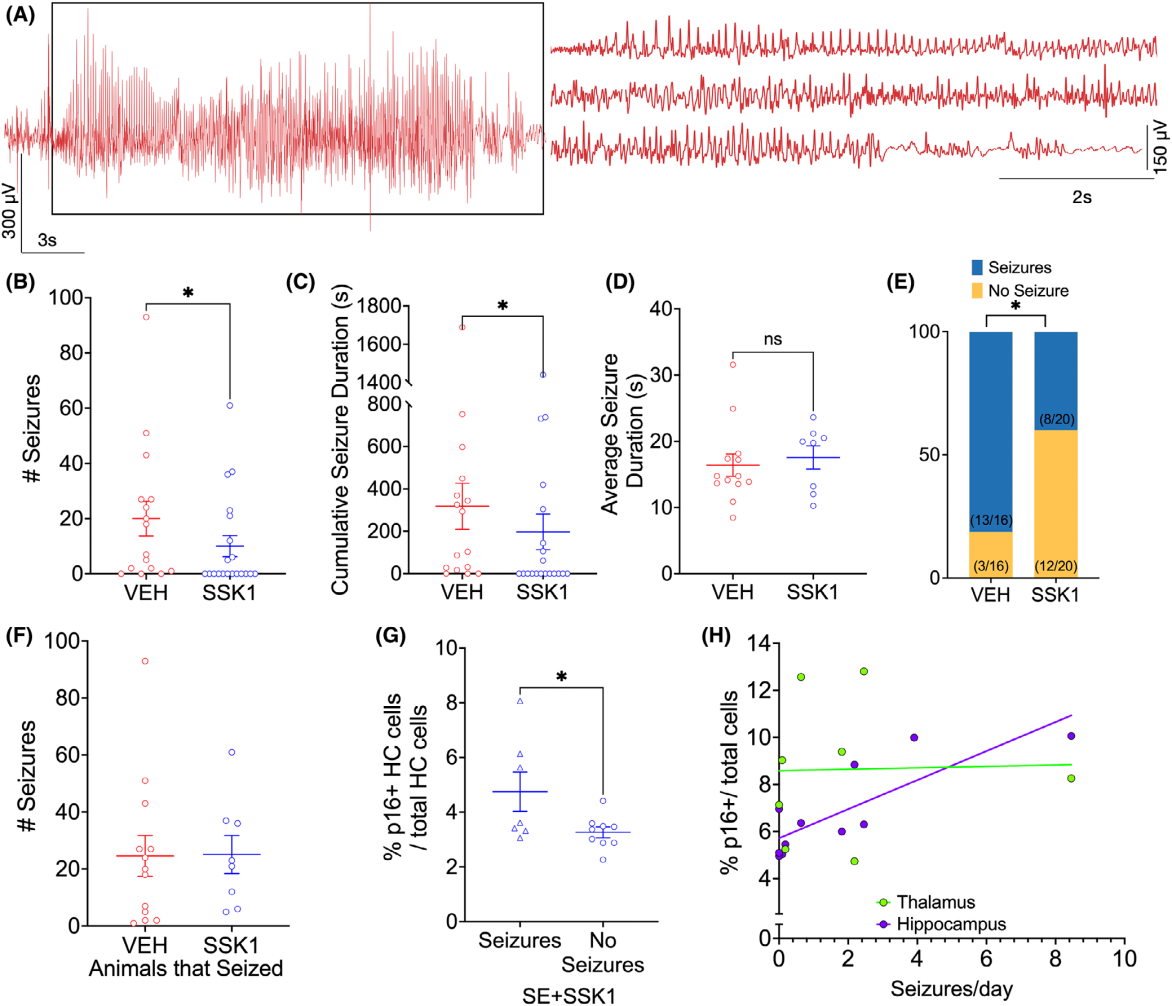
Selective senescence killing compound 1 (SSK1) treatment protects a majority of animals from seizures following SE. (A) A representative seizure. (B) SSK1 treatment significantly reduces the number of seizures recorded over an 11‐day monitoring period (*U* = 104, *p* = .0334, Mann–Whitney *U* test, one‐tailed). (C) SSK1 treatment significantly reduces the total time spent seizing over an 11‐day monitoring period (*U* = 104, *p* = .0334, Mann–Whitney *U* test, one‐tailed). (D) SSK1 treatment had no effect on the average duration of a single seizure event (*U* = 44, *p* = .5951, Mann–Whitney *U* test, two‐tailed). (E) Stacked bar showing the percentage of animals in each group that had at least one seizure (blue) or were seizure‐free (yellow) during the monitoring period. SSK1 treatment was significantly associated with seizure freedom (*p* = .0186, Fisher's exact test, two‐tailed). (F) When comparing only the animals with at least one recorded seizure in both groups, there is no difference in seizure burden (*U* = 45.50, *p* = .6573, Mann–Whitney *U* test, two‐tailed). (G) SSK1‐treated animals that were seizure‐free had significantly fewer hippocampal senescent cells (SCs) compared to SSK1 animals that had at least one seizure (*U* = 15, *p* = .0437, Mann–Whitney *U* test, one‐tailed). (H) Hippocampal SCs (purple), but not thalamic SCs (green), are significantly correlated with average seizures per day in VEH mice (Hippocampus: r_9_ = .7340, *p* = .0137; Thalamus: r_6_ = .2143, *p* = .4731, Spearman *r*, two‐tailed). Trend lines included for visual clarity. B, C: Vehicle (VEH; *n* = 16), SSK1 (*n* = 20). D, F: VEH (*n* = 13), SSK1 (*n* = 8). G: SSK1 Seizure (*n* = 7), SSK1 No Seizure (*n* = 9). H: Hippocampus (*n* = 11), Thalamus (*n* = 8). Mean ± SEM.

To test the effect of SSK1 treatment on spontaneous seizure outcomes, 4 months after SE, mice were implanted with wireless telemeters and recorded for 11 days. SSK1‐treated mice on average had fewer seizures during the monitoring period and a lower cumulative seizure duration compared to SE + VEH mice (Figure 4B,C). The average seizure duration, however, was similar between groups (Figure 4D). A majority (60%, 12/20) of SE + SSK1‐treated mice did not have a seizure during the monitoring period, a significantly greater fraction than SE + VEH mice (19%, 3/16) (Figure 4E). When we excluded animals that exhibited no seizures from our analysis of cumulative seizure burden, the SE + VEH and SE + SSK1 groups did not differ, indicating that the effect was driven by the animals completely protected from seizures (Figure 4F). Notably, the SSK1‐treated mice with one or more seizures during the observation period had elevated hippocampal SC counts compared to those with no seizures (Figure 4G). We found that, in SE + VEH mice, hippocampal p16+ cell burden was significantly correlated with seizure burden, but thalamic p16+ cell burden was not (Figure 4H).

Although unlikely, given that gemcitabine is not known to suppress seizures—and indeed has been anecdotally associated with increased risk for seizures^34^, ^35^—we sought to determine if SSK1 exerted an acute seizure‐suppressive effect. Four‐month‐old mice were pre‐treated with either SSK1 or VEH before treatment with the chemoconvulsant pentylenetetrazol. Neither evoked seizure severity nor latency to evoked seizures differed as a function of SSK1 treatment in this model (Figure S1), suggesting that SSK1 does not have acute seizure‐suppressive effects.

## 4 DISCUSSION

Our data demonstrate that SSK1 treatment significantly reduces seizure burden and behavioral comorbidities in SE mice. There is growing preclinical evidence that senolysis is a viable, disease‐modifying treatment for the epilepsies,^17^, ^31^, ^36^, ^37^ and our current findings add to this by demonstrating seizure protection with the potential for fewer off‐target effects than conventional senolytics.

SSK1, despite its intriguing and novel mechanism of action, is relatively under‐examined. SSK1 has been reported to have minimal impact on blood chemistry, liver enzymes, renal function, and peripheral cell apoptosis.^25^, ^27^ Only one other study has used SSK1 in the context of CNS disease: Ji and colleagues^27^ found that intravenous SSK1 formulated in nanoparticles reduced amyloid precursor protein levels in a mouse model of Alzheimer's disease. Nanoparticle treatment reduced p21, a cyclin‐dependent kinase inhibitor and senescence marker similar to p16; reduced amyloid levels; and improved performance in the Morris water maze.^27^ By contrast, when SSK1 was injected in a standard (non‐nanoparticle based) formulation, amyloid levels were decreased, but p21 levels and water maze performance were unchanged. The rationale for using nanoparticles was to improve the central delivery of SSK1, given the limited brain penetrance of its parent molecule, gemcitabine. This differs from our findings, which demonstrate clear target engagement (i.e., a reduction of SCs) by SSK1 following SE. It is plausible that, given the extensive and long‐lasting impairment of the blood–brain barrier in the pilocarpine model,^38^ SSK1 entry to the brain may have been increased. Notably, the ~45% reduction in SCs we observed with SSK1 is similar in magnitude to other studies using a range of senolytic compounds in a range of disease models.^12^, ^17^, ^25^, ^27^, ^39^

SSK1 treatment protected mice from SE‐induced deficits in hippocampal‐dependent object locational memory and navigational memory, but not object memory or anxiety‐like behaviors, which depend on extra‐hippocampal substrates.^40^, ^41^, ^42^ We found similar results in previous studies using both a genetic strategy and other senolytic drugs to ablate SCs.^17^, ^31^ We previously reported that senolysis improves hippocampal LTP post SE, and others have demonstrated that senolysis invigorates hippocampal neurogenesis in normal aging.^4^, ^39^ Here, we focused our histological analysis on the hippocampus and thalamus, but it is likely that the accumulation of SCs is not uniform throughout the brain and that SC accumulation in different brain regions produces differing behavioral phenotypes. Consistent with this, we found that in SE + VEH mice, SC number in the hippocampus, but not the thalamus, correlates with seizure burden.

The proportion of animals fully protected from seizures following SSK1 treatment is nearly double what we found in our prior studies using both genetic ablation and D + Q treatment.^17^ SSK1 treatment led to a bimodal effect: animals were either fully protected from seizures or there was no impact on total number of seizures. Understanding the mechanism(s) underlying the separation between these groups is a clear direction for future research. We recorded during a single time window, and for only 11 days. It thus remains unknown if ablation of SCs truly halts epileptogenesis or simply delays it beyond the period we recorded. However, we think this unlikely, as we recorded seizures 4 months after SE, a time at which spontaneous seizures are reliably reported in this model. Our data suggested—on the basis of acute PTZ‐evoked seizures—that SSK1 does not have acute anti‐seizure effects. However, the possibility remains, if unlikely, that SSK1 may impact the duration of SE or directly suppress the expression of spontaneous seizures (rather than modifying the disease process). Future studies using longitudinal EEG recordings across the study period would shed light on both effective treatment windows as well as the trajectory of effect of senolysis on epileptogenesis.

Although the protective effect of SC ablation strongly suggests directionality (i.e., that SCs drive seizures), it is plausible—and perhaps likely—that this effect is bidirectional, that is, that seizures also drive SC accumulation. Previously we observed SC accumulation as early as 14 days post SE,^17^ typically before we observe recurrent seizure activity. In the present study, we found that SSK1‐treated animals that developed epilepsy had significantly higher SC counts compared with seizure‐free counterparts. In a prior study, we reported that SC ablation protected against aging‐induced increased sensitivity to SE, suggesting that SCs may impact excitability/seizure threshold. We previously compared tissue from individuals who were seizure‐free after surgery (Engel Class 1) to tissue from individuals who continued to have seizures (Engel 2+). Hippocampal SC counts were significantly lower in the Engel 1 group as compared to the Engel 2+ group.^17^

The past decade has seen a rapid development of potential senolytic agents, acting through a wide range of mechanisms of action.^43^, ^44^, ^45^, ^46^, ^47^ Protective effects against normal aging, as well as from a range of disease phenotypes, have been reported across these mechanisms. Moreover, highly selective genetic approaches further support the notion that senolysis—and not off‐target effects—underlie the protection that has been observed.^12^, ^48^, ^49^ Delivery of these drugs is becoming more targeted, with galactose‐modified prodrugs^25^, ^45^ and antibody‐drug conjugates.^46^, ^47^ As a result, the senolytic toolkit continues to grow in application and flexibility. Here, we have found that SSK1, a newer targeted senolytic, protects against seizures and cognitive impairments following SE in mice. This adds to prior findings from us, and from others, suggesting that senotherapy may be a viable disease‐modifying treatment in epilepsy.

## CONFLICT OF INTEREST STATEMENT

None of the authors have any conflicts of interest to disclose. We confirm that we have read the Journal's position on issues involved in ethical publication and affirm that this report is consistent with those guidelines.

## Supporting information


Figure S1.

## Data Availability

The data that support the findings of this study are available from the corresponding author upon reasonable request.
