# Supplementary material for: Targeted senotherapy improves electrographic and behavioral outcomes in a mouse model of temporal lobe epilepsy
Source: Epilepsia. 2026 Apr 13;67(7):3779–90. doi: 10.1002/epi.70226 (PMC13360942; doi:10.1002/epi.70226)
Supplement: Supplementary file 1 — Figure S1. [file EPI-67-3779-s001.docx]

Supporting Information for

*Targeted senotherapy improves electrographic and behavioral outcomes in a mouse model of temporal lobe epilepsy.*

Supporting Methods:

Pentylenetetrazole (PTZ) evoked seizures

Four-month-old C57Bl/6J mice were pre-treated with a single injection of SSK1 (0.5mg/kg) or VEH (90% PBS, 5% Tween-80, 5% polyethylene glycol), i.p.. 15 minutes after pre-treatment, animals were placed in individual, empty cages and were administered 80mg/kg PTZ, s.c. Pretreatment timing was selected such that PTZ administration would overlap with the peak plasma concentration of SSK1, using gemcitabine as a reference (Shipley et al., 1992). Latency to behavioral changes, unilateral forelimb clonus, and tonic-clonic seizures, defined as bilateral forelimb clonus paired with either loss of balance or running/jumping, were recorded, with timing beginning immediately following injection. Monitoring was performed blind to treatment group.

Figure S1. SSK1 did not affect evoked seizures. **(A)** SSK1 did not alter the latency to PTZ-evoked seizures (Main Effect of Seizure Severity: F_1.834, 21.09_=180.4; p<0.0001; No Effect of Pretreatment: F_1,12_=0.2156; p=0.6507; No Interaction: F_1.834, 21.09_=0.1715; p=0.8257; Mixed-effects model with Geisser-Greenhouse correction). **(B)** All mice exhibited generalized tonic-clonic seizures. A,B: VEH (n=7), SSK1 (n=7). Mean$\pm$ S.E.M.

Shipley, L. A., Brown, T. J., Cornpropst, J. D., Hamilton, M., Daniels, W. D., & Culp, H. W. (1992). Metabolism and disposition of gemcitabine, and oncolytic deoxycytidine analog, in mice, rats, and dogs. *Drug Metabolism and Disposition: The Biological Fate of Chemicals*, *20*(6), 849–855.
